# Use of miRNA Sequencing to Reveal Hub miRNAs and the Effect of miR-582-3p/SMAD2 in the Progression of Hepatocellular Carcinoma

**DOI:** 10.3389/fgene.2022.819553

**Published:** 2022-03-21

**Authors:** Yi Zhao, Meizhang Li, Nana Miao, Wei Wei, Yulong Dong, Chenjie Tao, Jinzhong Chen, Yongyan Pei, Lieping Guo

**Affiliations:** ^1^ Department of Gastrointestinal Endoscopy, Eastern Hepatobiliary Surgery Hospital,The Third Hospital Affiliated of Naval Medical University, Shanghai, China; ^2^ Department of Oncology/Hematology, Eastern Hepatobiliary Hospital, Third Affiliated Hospital of Navy Military Medical University, Shanghai, China; ^3^ State Key Laboratory of Genetic Engineering, School of Life Sciences, Fudan University, Shanghai, China; ^4^ School of Medicine and Chemical Engineering, Guangdong Pharmaceutical University, Zhongshan, China

**Keywords:** miRNA sequencing, hepatocellular carcinoma, miR-582-3p/SMAD2, proliferation, migration and invasion

## Abstract

Hepatocellular carcinoma is a common tumor with a high fatality rate worldwide, and exploring its pathogenesis and deterioration mechanism is a focus for many researchers. Increasing evidence has shown that miRNAs are involved in the occurrence and progression of a variety of cancers, including hepatocellular carcinoma. Therefore, this study mainly aimed identify key miRNAs related to hepatocellular carcinoma and explore their potential functions and clinical significance. In this study, we performed miRNA sequencing on three pairs of hepatocellular carcinoma tissue samples and screened 26 differentially expressed miRNAs. Then 2 key miRNAs (miR-139-5p and miR-582-3p) were screened by Kaplan-Meier curve analysis, Cox multivariate analysis and qPCR methods. The expression of miR-582-3p was positively correlated with clinicopathological parameters in patients with hepatocellular carcinoma. Subsequently, miRwalk and starbase were used to predict the target genes of key miRNAs, and then the key pairs miR-582-3p/SMAD2 identified by WGCNA, PPI, qPCR and Pearson correlation analysis. Finally, a dual luciferase experiment, the rescue-of-function experiment and qPCR confirmed that miR-582-3p directly targets SMAD2 and regulates the proliferation, migration and invasion of HepG2 cells by targeting SMAD2. At the same time, interference with SMAD2 can influence the effect of miR-582-3p on HepG2 cells. In conclusion, our findings confirm that miR-582-3p is an independent factor for the prognosis of hepatocellular carcinoma patients, and can regulate the progression of hepatocellular carcinoma cells by targeting SMAD2.

## Introduction

Hepatocellular carcinoma is one of the most common cancers in the world, accounting for the second highest cancer mortality worldwide. Its pathogenesis and pathogenic genes are still the focus of current research (Hartke et al., 2017; Forner et al.,2018). Currently, surgery, ablation and liver transplantation are potential treatment options for hepatocellular carcinoma patients (Hartke et al.,2017; Grandhi et al.,2016). However, these options are only effective at the early stage of hepatocellular carcinoma. In the end-stage or recurrence of hepatocellular carcinoma, the average overall survival of patients is very short (Jan et al.,2019). Therefore, it is necessary to screen the influencing factors of hepatocellular carcinoma and understand the molecular mechanism of hepatocellular carcinoma progression to predict and control the disease as soon as possible.

MicroRNAs (miRNAs) are a class of small endogenous noncoding RNAs. Aberrant expression of miRNAs can affect the biological processes of various cancers and is mainly realized by binding the 3-UTR of target mRNA to regulate the expression of the target mRNA (Li et al., 2020; Nagy et al.,2018; Xu et al., 2020; Liu et al., 2020; Xin et al., 2020). In previous studies, many data have confirmed that miRNAs play an important role in regulating the development of hepatocellular carcinoma (Li et al,2020). It has been reported that miRNAs regulate the proliferation, differentiation, apoptosis, invasion and metastasis of hepatoma cells by binding to mRNA (Ji et al., 2019). In addition, the expression of miRNAs is also related to the prognosis and clinicopathological parameters of hepatocellular carcinoma (Qin et al,2019). These results indicate that miRNAs can be used as potential biomarkers for the diagnosis or treatment of hepatocellular carcinoma. At present, the search for miRNA biomarkers is still focused on a single method of bioinformatics analysis, which makes it difficult to represent the complex interaction and functional association between genes in the process of hepatocarcinogenesis. Therefore, there is a lack of a comprehensive and systematic research strategy to show the interaction between miRNAs and mRNAs in hepatocellular carcinoma.

This study aims to study the interaction between mRNA and miRNAs, to screen the key miRNAs involved in the occurrence and development of liver cancer and identify new indicators that affect the progression of hepatocellular carcinoma. We defined the miR-582-3p/SMAD2 regulatory axis through miRNA sequencing, bioinformatics analysis, weighted gene co-expression network (WGCNA), PPI, correlation analysis, qPCR, RNA interference and dual luciferase experiments. Subsequently, a series of methods were used to further explore the potential functions and clinical significance of the miR-582-3p/SMAD2 regulatory axis.

## Materials and Methods

### Clinical Sample Collection

Hepatocellular carcinoma tissues and adjacent tissues were collected from 23 hepatocellular carcinoma patients at the Third Affiliated Hospital of Naval Military Medical University (3 pairs of samples were used for miRNA sequencing, and the rest were used for qPCR experiments) (Supplementary Material S1). This study was approved by the Research Ethics Committee of the Third Affiliated Hospital of Naval Military Medical University. All patients signed informed consent forms in accordance with the Declaration of Helsinki. All patients were recruited according to histopathological evaluation, and did not receive radiotherapy or chemotherapy before surgery. Tissues samples were immediately frozen in liquid nitrogen or stored at − 80°C after the surgery.

### MiRNA Sequencing

Total RNA was isolated using the German Qiagen RNEasy Mini kit (Germany, Qiagen). Then Nanodrop 8,000 was used to detect the concentration and purity of the RNA. 1μg total RNA was used as the starting amount, and then the Truseq™ Small RNA Sample Prep Kit (United States, Illumina) was used to connect the 3′ end adapter and the 5’ end adapter and invert them into cDNA. The library was then enriched by PCR and purified using a 6% Novex TBE PAGE gel (United States, Invitrogen). Subsequently, TBS380 Picogreen (United States, Invitrogen) was used for quantification, and cBot Truseq SR Cluster Kit v3 (United States, Illumina) was used for bridge PCR amplification. Finally, SE50 sequencing was performed. The library was constructed and sequenced by Shanghai Yuanshen Biomedical Technology Co., Ltd.

### Data Processing and Identification of Differentially Expressed miRNAs

First, quality control was performed on raw reads: 1) The bases with lower sequencing quality at the 3′end (quality value less than 20) were removed; 2) The linker sequence in the reads was removed, and sequences lacking inserts due to linker self-connection and other reasons were removed. 3) Reads with an N ratio of more than 10% were removed; 4) Shorter reads (<18 nt) were removed; 5) Sequences with a length of 18–32 nt were extracted. Then, based on the clean reads, bioinformatics analysis was carried out. Unique sequences were obtained by combining reads with exactly the same sequence. Then, the Rfam (http://Rfam.sanger.ac.uk/) database was used to annotate the measured small RNAs and remove non-miRNA sequences, such as rRNAs, scRNAs, snoRNAs, snRNAs and tRNAs. Finally, the results were compared with the human miRNA precursor and mature body sequences in the miRBase database (http://www.mirbase.org/).

The Limma package was used to screen the differentially expressed miRNAs in the miRNA sequencing data between hepatocellular carcinoma tissues and normal tissues. The screening criteria were: |log2 (fold-change)|>1, false discovery rate (FDR) < 0.05 and p < 0.05. Then, the pheatmap and ggplot2 packages to draw a heatmap and a volcano map of the differentially expressed miRNAs.

### Kaplan–Meier Survival Curve Analysis

To investigate the impact of the expression levels of differentially expressed miRNAs on the prognostic survival of hepatocellular carcinoma patients, Kaplan–Meier survival curve analysis was performed using the online tool “starbase (http://starbase.sysu.edu.cn/). The statistical significance was set at *p* < 0.05.

Construction of the miRNA Cox proportional risk regression model.

The GSE31384 dataset expression profile and clinical information were downloaded from the GEO database. Then sangerbox (http://sangerbox.com/Index) was used to perform Cox multivariate regression analysis on the results of the survival analysis. Then, based on Cox multifactor analysis, we built a Cox proportional risk assessment model and obtained the formula: β_miRNA1_×expression of miRNA1+β_miRNA2_×expression of miRNA2 + ……+ β_miRNAn_× expression of miRNAn, where β represents the Cox multifactor regression coefficient. Then, the risk value was calculated according to the formula, and the sample was divided into high and low-risk groups. Finally, the survival analysis was performed on the two groups, and the 1-, 3-, and 5-year survival receiver operating characteristic (ROC) curves were drawn to test the feasibility of the model’s predictive ability.

### Weighed Gene Co-expression Network Analysis

The GSE57958 mRNA expression profile was downloaded from the GEO database. Then, the WGCNA package was used to construct a gene co-expression network. Subsequently, we set the parameters as follows: height = 0, net type = unsigned, block size = 20,000" to obtain the best soft threshold. Next, we set the parameters as: lock size = 7,000, min Module size = 30, deep split = 2, merge cut height = 0.35, and hub cut = 0.9 to construct a topological overlap matrix (TOM). Finally, we performed hierarchical clustering to identify gene modules and used Pearson correlation to analyze the co-expression module and clinical characteristics to obtain the hub co-expression module.

### Gene Function Analysis

In order to further analyze the biological functions of the screened mRNAs, we used David online tools (http://david.abcc.ncifcrf.gov/) and Metascape (http://metascape.org/gp/index.html) to perform Gene Ontology (GO) and Kyoto Encyclopedia of Genes and Genomes (KEGG) analysis. p < 0.05 was considered statistically significant.

### Protein–Protein Interaction Network and Key mRNA Selection

The STRING online database (http://string-db.org) was used to identify and construct PPI network interactions. An interaction confidence of 0.4 was considered significant. Then the results were imported into Cytoscape for visualization. Subsequently, the MCODE plug-in was used to screen the PPI core network.

### Reverse Transcription-Quantitative Polymerase Chain Reaction Analysis

Total RNA was extracted using the RNeasy Mini Kit (Qiagen, United States) according to the manufacturer’s protocol. Then the extracted RNA was qualitatively controlled and quantified by Nanodrop. For mRNA, the equivalent amount of RNA was reverse transcribed into cDNA with Golden star ™ RT6 cDNA synthesis kit ver.2 (TsingKe, China). mRNA expression was then examined by qRT-PCR with Master qPCR Mix (SYBR GREEN 1) (TsingKe, China). GAPDH expression was evaluated as an endogenous control. The primers were designed using Primer 5.0 software (Supplementary Material S2) and synthesized by Sangon Biotech (Shanghai, China). For miRNA, RNA reverse transcription and expression detection were performed by using a Bulge-Loop miRNA qRT-PCR Starter Kit (Ribobio, China). U6 expression was evaluated as an endogenous control. The primers were purchased from Ribobio (miR-582-3p: MQPS0001915-1-100; U6: miRAN0002-1-100). The 2^−ΔΔCt^ method was used to calculate the levels of expression. Excel was used to analyze the qRT-PCR data, and each reaction was performed in triplicate. The two groups were analyzed using *t*-test (*p* < 0.05).

### Cell Culture and Transfection

HepG2 cell lines were purchased from the Beijing Beina Chuanglian Biotechnology Research Institute (Beijing, China). The cells were cultured in Dulbecco’s modified Eagle’s medium (Invitrogen, United States) supplemented with 10% fetal bovine serum (Invitrogen, United States), 100 U/ml penicillin (Sigma, United States) and 100 μg/ml streptomycin (Sigma, United States) under a humidified atmosphere of 5% CO_2_ at 37°C. MiR-582-3p mimics (miR10004797-1-5) and miR-582-3p inhibitor (miR20004797-1-5) were purchased from Ribobio (China) and transfected into HepG2 cell lines at a final concentration of 100 nM using Lipofectamine RNAiMAX (Invitrogen, United States) for 24 h at 37 °C in accordance with the manufacturer’s instructions. Moreover, pCDH-SMAD2 and si- SMAD2 (siG0812151563701-1-5) were also purchased from Ribobio (China) and transfected into HepG2 cell lines using Lipofectamine RNAiMAX (Invitrogen, United States) for 24 h at 37°C in accordance with the manufacturer’s instructions.

### Luciferase Reporter Gene Assay

The wild-type (WT) and mutant-type (MT) 3′-UTRs of SMAD2 (pmiR-RB-Report™-SMAD2) were constructed by Ribobio (China). Each target fragment was inserted into the pmiR-RB-Report™ vector (Ribobio, China). Fluorescence intensity was measured using the Dual-Luciferase Reporter^®^ Assay System (Promega, United States) on a multifunctional microplate reader (Thermo Fisher, United States).

### Cell Counting Kit-8

Logarithmic growth phase HepG2 cells were seeded in 96-well plates with 5,000 cells per well. After 24 h of transfection, CCK-8 reagent (10 μL per well, Beyotime, China) was added and incubated for 2 h, and then the absorbance was measured at 450 nm with a multifunctional microplate reader (Thermo, United States). Each experiment was performed three times.

### Migration and Invasion Assay

For transwell migration test, HepG2 cells were digested by trypsin, and then placed the digested cell suspension in the upper chamber of each insert (Corning, United States) containing the noncoated membrane. The lower cavity was supplemented with 600 μL 1% fetal bovine serum (Gibco, United States). After incubating at 37°C for 24 h, the upper surface of the membrane was removed with a cotton swab, and the lower surface was stained with 0.1% crystal violet for 30 min. For invasive tests, a chamber containing Matrigel (BD, United States) was used. Matrigel was diluted with serum-free DMEM (volume ratio was 1:3) and dissolved at 4°C overnight. The 40 μL mixture was uniformly added to the precooled Transwell chamber and incubated in an incubator at 37°C for 2 h to solidify Matrigel. HepG2 cells transfected for 24 h were collected and single cell suspension was prepared. 1 × 10^5^ cells were added to the upper chamber (Corning, United States) containing matrix gel. DMEM containing 20% fetal bovine serum was added into the lower chamber. After 24 h, the upper chamber was removed, and the cells were fixed with 4% paraformaldehyde (Beyotime, China) after PBS rinsing, and stained with 1% crystal violet (Beyotime, China). Cell count was observed under light microscope.

### Statistical Analysis

All the data are presented as the means ± SD (standard deviation) and the statistical analyses were performed by SPSS and GraphPad Prism. The t test was used to evaluate the significant difference between the two groups. One-way analysis of variance (ANOVA) was used to compare multiple groups. A *p* < 0.05 was considered statistically significant.

## Results

### Overview of the miRNA Sequencing Results

**FIGURE 1:**
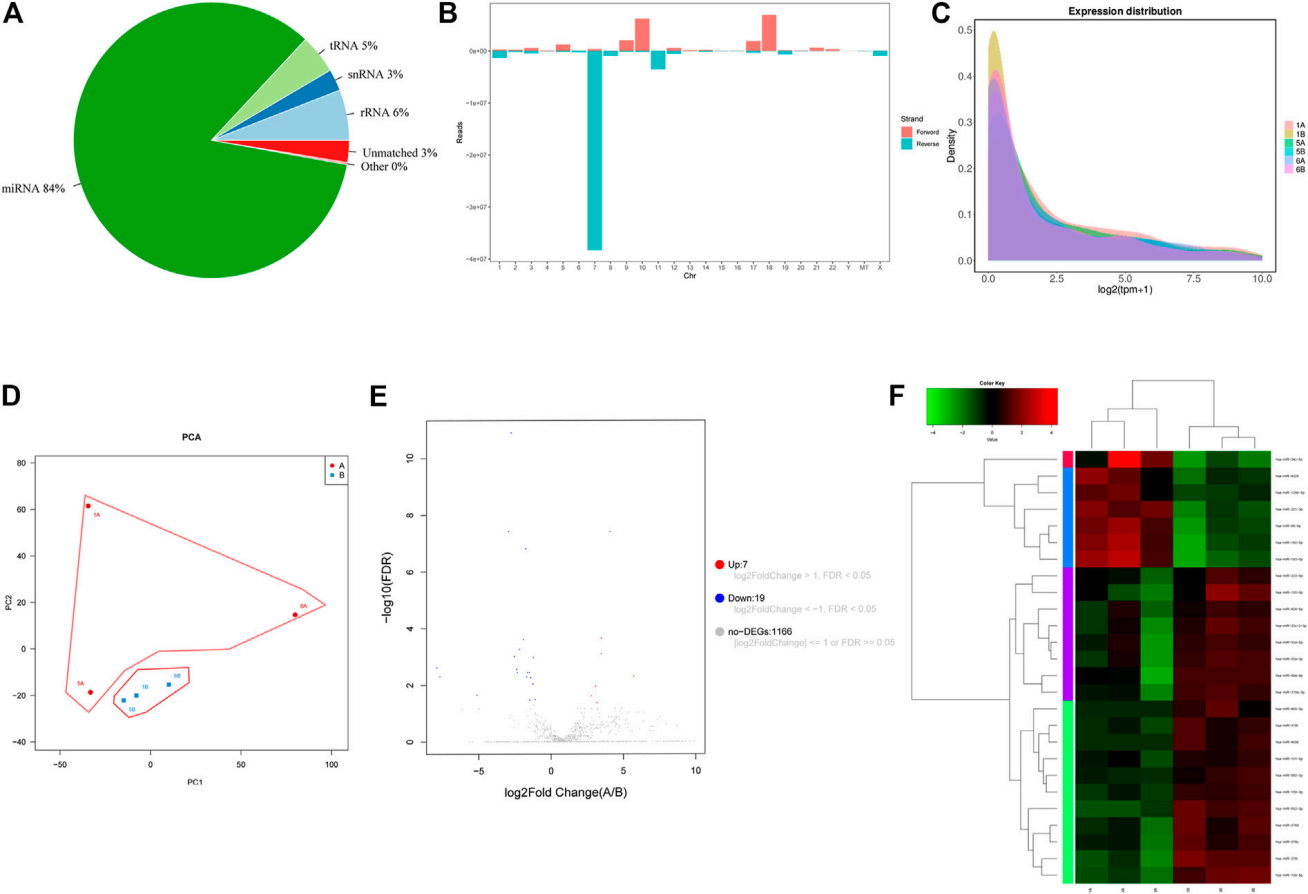
**(A)** 2D pie diagram of the number/total amount of common and unique sequences between samples. **(B)** The clean reads of all samples and the reference genome comparison results are displayed. **(C)** Distribution of the expression density of all samples. **(D)** PCA diagram of sample expression. **(E)** Volcano plot of the differential expression profiles of miRNAs. The blue dots represent downregulated miRNAs, and red dots represent upregulated miRNAs. **(F)** Heatmap of the differential expression profiles of miRNAs. “A”: hepatocellular carcinoma. “B”: adjacent tissue.

Small RNA sequencing was performed on three hepatocellular carcinoma tissues and three adjacent tissues. Approximately 7,2852061 raw reads were obtained from the six libraries. Then, all low-quality reads were removed, leaving only reads with a size between 18-32 nucleotides. Finally, approximately 68355381 clean reads were obtained (Table 1). In addition, 97.41% of these clean reads were successfully matched to the Rfam database. In these matched reads, the most abundant reads were miRNA (84.11%), and the rest were mainly rRNA (5.93%), snRNA (2.53%), tRNA (4.59%) and other unknown RNA molecules (0.25%) (Figure 1A). In addition, the small RNA sequences were located on the reference genome, and the distribution of these sequences on the genome was analyzed. The genome mapping rate was 98.97%, indicating that there were few impurities and the reads were of high quality (Supplementary Material S3). As shown in Figure 1B, the antisense strand of chr-7 was the most distributed, followed by the sense strands of chr-10 and chr-18. Subsequently, we analyzed the expression density of each sample, and the results showed that the expression pattern of each sample was essentially the same, indicating that the data could be compared and analyzed (Figure 1C). The PCA results showed that the tumor and the normal group could be distinguished well (Figure 1D).

**TABLE 1:** Raw data statistics of miRNA sequencing.

Sample	Raw_reads	Adapter_only	N_reads	<18 nt	>32 nt	Clean_reads
1A	2464538	4211	520	122750	161636	2175421
1B	17849283	4937	4342	519451	357147	16963406
5A	5721012	4669	1417	202666	278532	5233728
5B	10969971	4497	2630	144392	858612	9959840
6A	15852351	5563	4016	295651	263084	15284037
6B	19994906	6895	4792	884544	359726	18738949

A, hepatocellular carcinoma; B, adjacent tissue.

### Screening of Differentially Expressed miRNAs in Hepatocellular Carcinoma Tissues

By comparison with the miRbase-posited sequence, we identified 2,948 miRNAs of 589 miRNA families (Supplementary Material S4). Then we set the screening criteria: |log2FC| ≥ 1 and FDR-adjusted p < 0.05, screening differentially expressed miRNAs from identified miRNAs. Based on these criteria, 26 miRNAs (7 up-regulated and 19 down-regulated) exhibited differential expression between hepatocellular carcinoma and normal tissues (Table 2). The volcano map illustrated in Figure 1E shows the expression levels of all screened miRNAs. Cluster analysis also revealed that the miRNA expression patterns between hepatocellular carcinoma tissues and normal tissues were clearly distinct (Figure 1F).

**TABLE 2:** Differentially expressed miRNAs in adjacent tissue and hepatocellular carcinoma tissues.

Symbol	logFC	Padj	Pvalue	OS- pValue
**Up regulation**				
hsa-miR-183-5p	4.06726	3.87E-08	2.22E-10	0.180
hsa-miR-182-5p	3.47758	2.24 E-04	2.14E-06	0.400
hsa-miR-96-5p	3.46278	8.02 E-04	1.23E-05	0.250
hsa-miR-34c-5p	5.70973	4.99 E-03	1.55 E-04	0.390
hsa-miR-221-3p	3.08350	1.11 E-02	4.47 E-04	0.053
hsa-miR-582-3p	2.77700	2.42 E-02	1.06 E-03	0.043^a^
hsa-miR-4326	3.17610	4.27 E-02	2.12 E-03	0.790
**Down regulation**2				
hsa-miR-4686	−7.9028	2.42E-03	5.08E-05	0.260
hsa-miR-490-3p	−7.6888	5.00E-03	1.62E-04	0.250
hsa-miR-552-3p	−5.1247	2.24E-02	9.42E-04	0.380
hsa-miR-378i	−2.9372	3.80E-08	1.45E-10	0.044^a^
hsa-miR-139-5p	−2.7657	1.26E-11	2.41E-14	3.4E-06^a^
hsa-miR-139-3p	−2.5419	9.80E-04	1.69E-05	0.001^a^
hsa-miR-150-5p	−2.3852	2.78E-03	6.39E-05	0.120
hsa-miR-378f	−2.3369	3.57E-03	1.02E-04	0.700
hsa-miR-378d	−2.1825	5.49E-04	7.35E-06	0.420
hsa-miR-378c	−1.9091	2.47E-04	2.83E-06	0.600
hsa-miR-378a-3p	−1.7573	1.54E-07	1.18E-09	0.410
hsa-miR-30c-2-3p	−1.6862	5.15E-03	1.77E-04	0.150
hsa-miR-99a-5p	−1.6154	3.57E-03	9.64E-05	0.062
hsa-miR-30a-3p	−1.5058	3.57E-03	9.45E-05	0.580
hsa-miR-1296-5p	−1.4721	3.38E-02	1.62E-03	0.480
hsa-miR-223-5p	−1.4076	5.51E-03	2.00E-04	0.950
hsa-miR-30a-5p	−1.2598	9.13E-03	3.49E-04	0.330
hsa-miR-101-3p	−1.2289	1.08E-03	2.06E-05	0.006^a^
hsa-miR-424-5p	−1.1063	3.23E-02	1.48E-03	0.110

a means statistically significant.

“OS” overall survival.

### Prognostic Analysis of Differentially Expressed miRNAs

**FIGURE 2:**
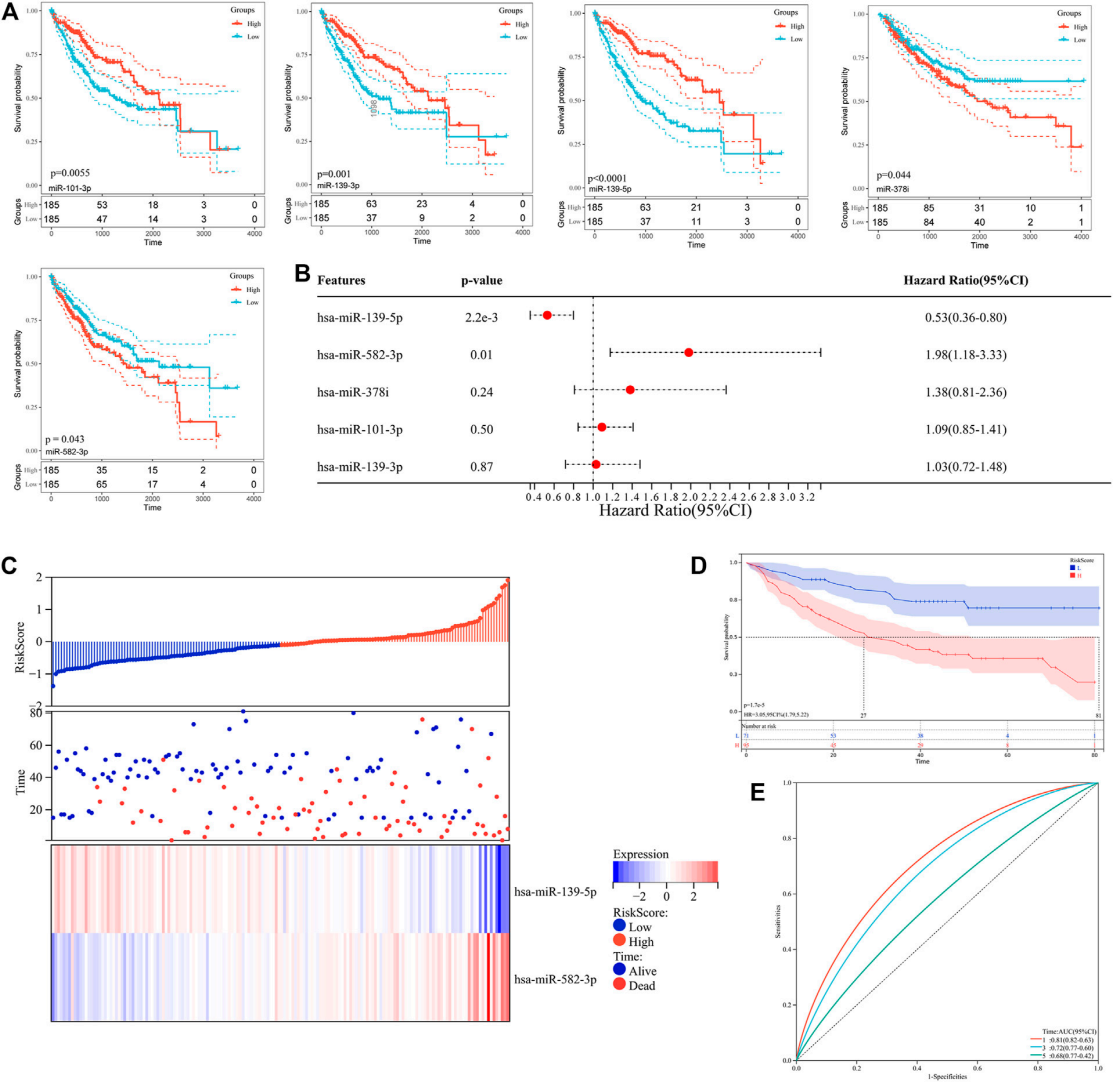
**(A)** Kaplan–Meier survival curve analysis of five miRNAs (miR-139-5p, miR-139-3p, miR-101-3p, miR-582-3p, miR-378i). **(B)** Forest plot of Cox multivariate analysis of five miRNAs (miR-139-5p, miR-139-3p, miR-101-3p, miR-582-3p, miR-378i). **(C)** Relationship between the expression of miR-139-5p and miR-582-3p and patient survival in the risk assessment model. **(D)** Kaplan–Meier survival curve analysis in the risk assessment model. **(E)** ROC curve analysis in the risk assessment model.

To further assess the clinical relevance of differentially expressed miRNAs, the Kaplan-Meier database was used to evaluate the overall survival of hepatocellular carcinoma patients with high or low miRNA expression. As shown in Figure 2A, five miRNAs (miR-139-5p, miR-139-3p, miR-101-3p, miR-582-3p, miR-378i) were associated with the prognosis of hepatocellular carcinoma patients (Table 2). Low expression levels of miR-582-3p and miR-378i were associated with good prognosis in patients with hepatocellular carcinoma. However, low expression levels of miR-139-5p, miR-139-3p, and miR-101-3p were connected with poor prognosis of patients with hepatocellular carcinoma. Furthermore, we downloaded clinical survival information and miRNA expression profile (GSE31384) from GEO database, which included 166 clinical samples (Supplementary Material S5). Subsequently, we analyzed the survival relationship between prognosis-related miRNAs and hepatocellular carcinoma patients by Cox regression analysis. Multivariate Cox regression analysis revealed that 2 miRNAs (miR-139-5p and miR-582-3p) were closely related to the overall survival rate of hepatocellular carcinoma patients (Figure 2B). In addition, based on the Cox analysis data, we used miR-139-5p and miR-582-3p to construct a risk assessment model for expression and survival. The model formula was as follows: Risk Score = 0.533 × expression of miR-139-5p + 1.979 ×expression of miR-582-3p. Subsequently, the risk value of each sample was calculated according to the risk assessment formula, and the samples were divided into high and low-risk groups based on the median. The scatter plot and heatmap show that the change trend between the expression levels of the 2 miRNAs and the survival status is consistent with the survival analysis results (Figure 2C). In addition, Kaplan-Meier curve analysis was performed on the two groups, and the results showed that the prognosis of the high-risk group was worse than that of the low-risk group (Figure 2D). The ROC curve analysis results revealed that the areas under the curve (AUCs) for 1-, three- and five- years survivals were 0.81,0.72 and 0.68, respectively (Figure 2E). These data indicate that the risk model has a certain monitoring value and that miR-139-5p and miR-582-3p may be 2 independent prognostic factors for hepatocellular carcinoma.

### Target Prediction and Functional Enrichment Analysis of miRNA-Associated mRNAs in Hepatocellular Carcinoma

**FIGURE 3:**
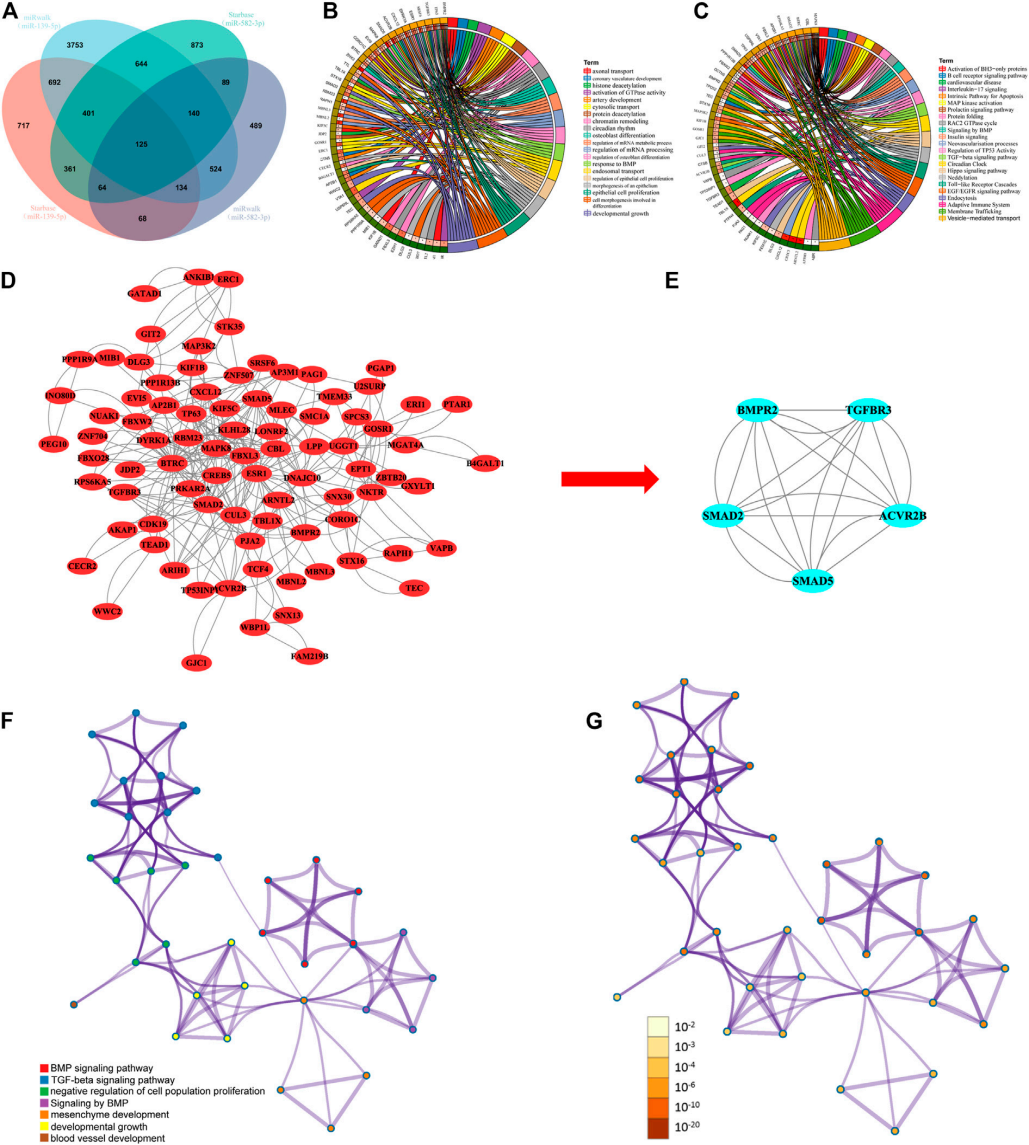
**(A)** Venn diagram showing the overlapping target genes of miR-139-5p and miR-582-3p. **(B)** and **(C)** GO and KEGG analyses of overlapping target genes. **(D)** PPI analysis of overlapping target genes. **(E)** Hub network screening in the PPI coexpression network by the MCODE plug-in. **(F)** and **(G)** Network of enriched terms: **(F)** colored by cluster ID, where nodes that share the same cluster ID are typically close to each other; **(G)** colored by p value, where terms containing more genes tend to have a more significant p value.

We predict the 2 prognostic miRNA (miR-139-5p, miR-582-3p) target genes using two independent online analysis tools (miRwalk and starbase). MiR-139-5p obtained 6,413 and 2,562 target genes, respectively. while miR-582-3p obtained 1,633 and 2,697 target genes, respectively. Through the Venn diagram comparison, 125 overlapping mRNAs were identified (Figure 3A). To further explore the biological functions of miR-139-5p and miR-582-3p, we performed GO and KEGG analyses on the 125 overlapping mRNAs. The GO results showed that these mRNAs were mainly enriched in epithelial cell proliferation, activation of GTPase activity, regulation of epithelial cell proliferation, cell morphogenesis involved in differentiation and cytosolic transport (Figure 3B). The KEGG analysis mainly revealed enrichments in the TGF-beta signaling pathway, the Hippo signaling pathway, endocytosis, vesicle-mediated transport and signaling by BMP (Figure 3C).

### Construction of PPI Networks for Overlapping mRNAs

To study the interactions between overlapping mRNAs, we used the STRING database to analyze the 125 overlapping mRNAs. The confidence score was set to ≥0.4, and the results were then visualized with Cytoscape. The network was found to contain 86 nodes and 270 edges (Figure 3D). Then, the MCODE plug-in was used to perform module analysis on the PPI network and define a sub-network (Figure 3E). The sub-network contained five nodes and 20 edges and was mainly involved in the negative regulation of cell population proliferation, blood vessel development, mesenchyme development, developmental growth, the TGF-beta signaling pathway and signaling by BMP (Figure 3F; Figure 3G).

### Weighed Gene Co-expression Network Analysis of mRNAs

**FIGURE 4:**
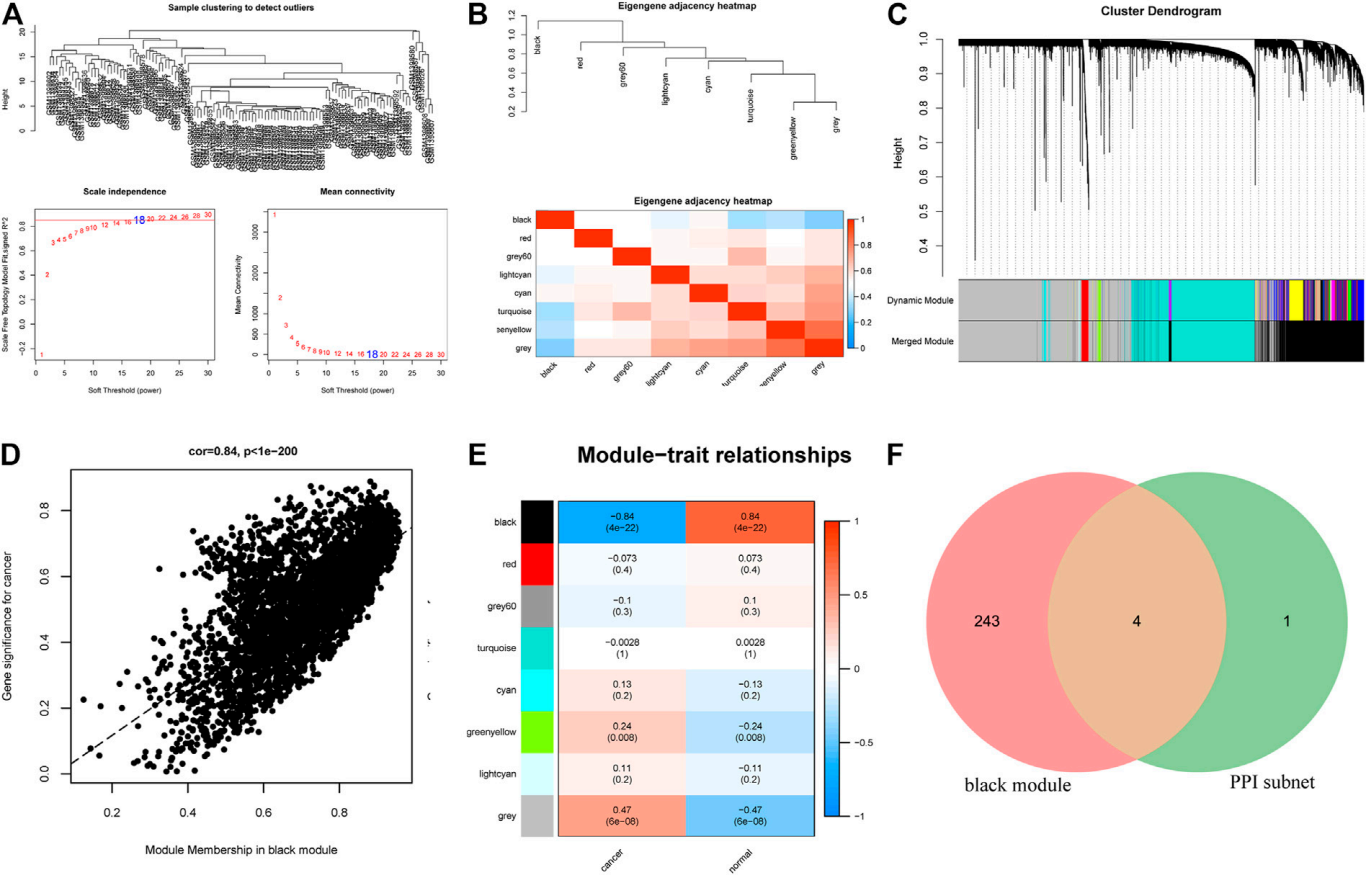
**(A)** Sample clustering results, soft threshold results and soft threshold and average connectivity. **(B)** The clustering relationship between WGCNA modules and modules. The upper part is the clustering tree between modules, and the lower part is the clustering heatmap between modules. **(C)** Dendrogram of all differentially expressed genes clustered based on a dissimilarity measure. **(D)** Correlation analysis between cancer and the black module. **(E)** Correlation analysis of phenotype and each module. **(F)** Venn diagram showing the overlapping mRNAs between the mRNAs in the WGCNA hub modules and the mRNAs in the PPI core sub-network.

To further screen the hub target genes of miR-139-5p and miR-582-3p, we downloaded the hepatocellular carcinoma RNA expression profile dataset GSE57958 from the CEO database. WGCNA was performed on 14,449 genes among them. The pick soft threshold was used to estimate the distributions of log (k/pk) coefficients and mean connectivity. According to the scale-free topology criterion, we chose 18 as the soft threshold power and the system clustering tree was constructed based on Pearson’s coefficients of each gene in the present study (Figure 4A; Figure 4B). The results revealed that these genes were classified into eight modules according to their correlation with traits (Figure 4C). We found that the black module eigengene containing 274 mRNAs had the highest correlation index with traits (r = 0.84, *p* = 4E-22, Figure 4E). The expression of these mRNAs in the black module was highly correlated with traits (Figure 4D). Thus, we obtained a hub module containing 274 mRNAs by WGCNA. Subsequently, the mRNAs in the black module and the PPI sub-network were compared through a Venn diagram, and four overlapping mRNAs (ACVR2B, SMAD2, SMAD5, and TGFBR3) (Figure 4F) were obtained. These four overlapping mRNAs may be the hub mRNAs involved in the progression of hepatocellular carcinoma.

### Verification of the Expression of Hub miRNAs and mRNAs in Hepatocellular Carcinoma Tissues by qPCR

**FIGURE 5:**
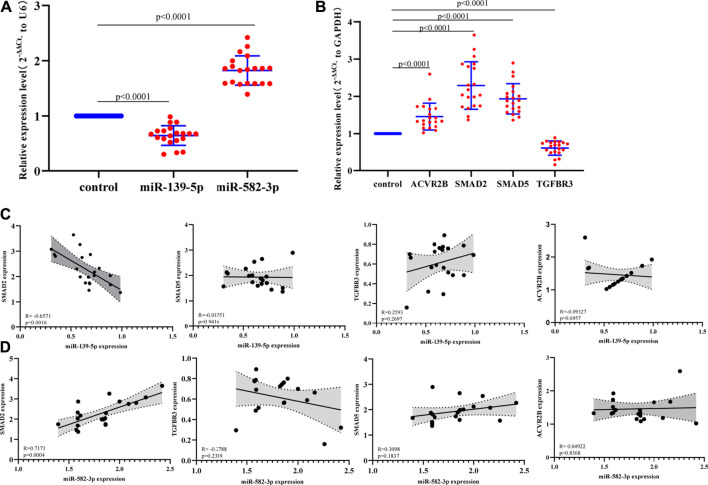
**(A)** Expression of miR-139-5p and miR-582-3p in hepatocellular carcinoma and normal tissues by qPCR. The data are shown as the mean ± standard deviation. **(B)** Expression profiles of four hub mRNAs (ACVR2B, SMAD2, SMAD5 and TGFBR3) in hepatocellular carcinoma and normal tissues by qPCR. The data are shown as the mean ± standard deviation. **(C)** Correlation analysis between miR-139-5p and the four hub mRNAs (ACVR2B, SMAD2, SMAD5, and TGFBR3). **(D)** Correlation analysis between miR-582-3p and the four hub mRNAs (ACVR2B, SMAD2, SMAD5, and TGFBR3).

We selected 2 differentially expressed miRNAs (miR-139-5p and miR-582-3p) and four hub mRNAs (ACVR2B, SMAD2, SMAD5, and TGFBR3) for qRT-PCR verification in 20 hepatocellular carcinoma tissues and 20 adjacent tissues (Supplementary Material S1). The results showed that miR-139-5p and TGFBR3 were significantly down-regulated in hepatocellular carcinoma tissues, and miR-582-3p, ACVR2B, SMAD2 and SMAD5 were up-regulated in hepatocellular carcinoma tissues (Figure 5A; Figure 5B). These results indicate that the expression patterns of miR-139-3p and miR-139-5p were consistent with those revealed by miRNA sequencing (Table 2). In addition, the expression patterns of the mRNAs were consistent with the results of biosynthesis analysis (Supplementary Figure S1). Subsequently, we analyzed the correlation between the expression levels of 2 miRNAs and four hub mRNAs (ACVR2B, SMAD2, SMAD5 and TGFBR3). The analysis results showed that miR-139-5p was negatively correlated with SMAD2 expression (Figure 5C). In contrast, miR-582-3p was also positively correlated with the expression level of SMAD2 (Figure 5D). However, there have been many studies on miR-139-5p in the progression and mechanism of hepatocellular carcinoma, while there have been relatively few studies on miR-582-3p in this area; therefore, miR-582-3p/SMAD2 was selected for follow-up studies in this project.

### Effect of miR-582-3p on the Progression of Hepatocellular Carcinoma Cells

**FIGURE 6:**
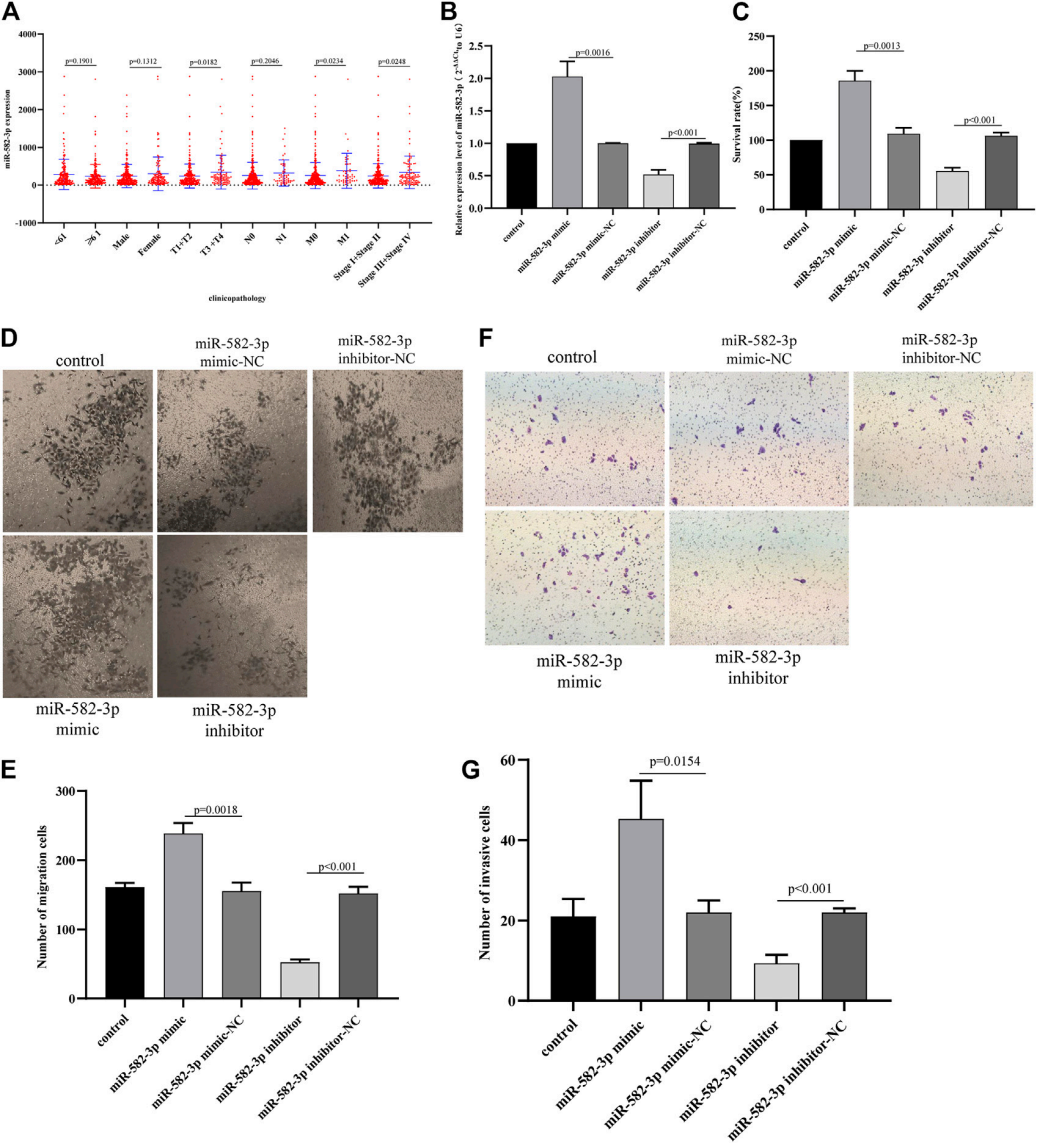
**(A)** Correlation between miR-582-3p expression and clinicopathological characteristics of hepatocellular carcinoma. **(B)** Changes in the expression of miR-582-3p in HepG2 cells after the addition of miR-582-3p mimics and inhibitor, as shown by qPCR. The data are shown as the mean ± standard deviation. **(C)** Analysis of the effects of miR-582-3p knockdown or overexpression on the proliferation of HepG2 cells by CCK-8 assay. **(D)** and **(E)** Analysis of the effects of miR-582-3p knockdown or overexpression on the migration of HepG2 cells by Transwell migration assay. **(F)** and **(G)** Analysis of the effects of miR-582-3p knockdown or overexpression on the invasion of HepG2 cells by Transwell assay.

First, we analyzed the relationship between miR-582-3p expression and clinical parameters in patients with hepatocellular carcinoma. The analysis results revealed that the expression of miR-582-3p was positively correlated with TNM staging (pathologic_T and pathologic_M) and tumor grade in patients with hepatocellular carcinoma (Figure 6A), indicating that the high expression of miR-582-3p is likely to cause tumor deterioration in patients. This finding is consistent with the survival analysis results of miR-582-3p. Subsequently, we further analyzed miR-582-3p through in vitro cell experiments. The miR-582-3p mimic and inhibitor were transfected into HepG2 cells, and then the transfection efficiency was determined by qPCR. The results showed that miR-582-3p expression was significantly increased in HepG2 cells after transfection with miR-582-3p mimic, while the expression of miR-582-3p in HEPG2 cells was significantly decreased after transfection with miR-582-3p inhibitor (Figure 6B). CCK-8 experiments showed that overexpression of miR-582-3p significantly enhanced the proliferation of HepG2 cells. In contrast, silencing miR-582-3p significantly reduced the proliferation activity of HepG2 cells (Figure 6C). Then we conducted a wound healing experiment to evaluate whether miR-582-3p also affects the horizontal migration of HepG2 cells. The results showed that the migration rate of HepG2 cells increased after miR-582-3p mimic transfection, while the migration rate of HepG2 cells decreased significantly after transfection with miR-582-3p inhibitor (Figure 6D; Figure 6E). We obtained similar results in transwell invasion experiments. Overexpression of miR-582-3p significantly promoted the invasion ability of miR-582-3p cells (Figure 6F; Figure 6G). In conclusion, miR-582-3p can regulate the proliferation, migration and invasion of hepatocellular carcinoma cells.

### SMAD2 Is a Direct Target of miR-582-3p

**FIGURE 7:**
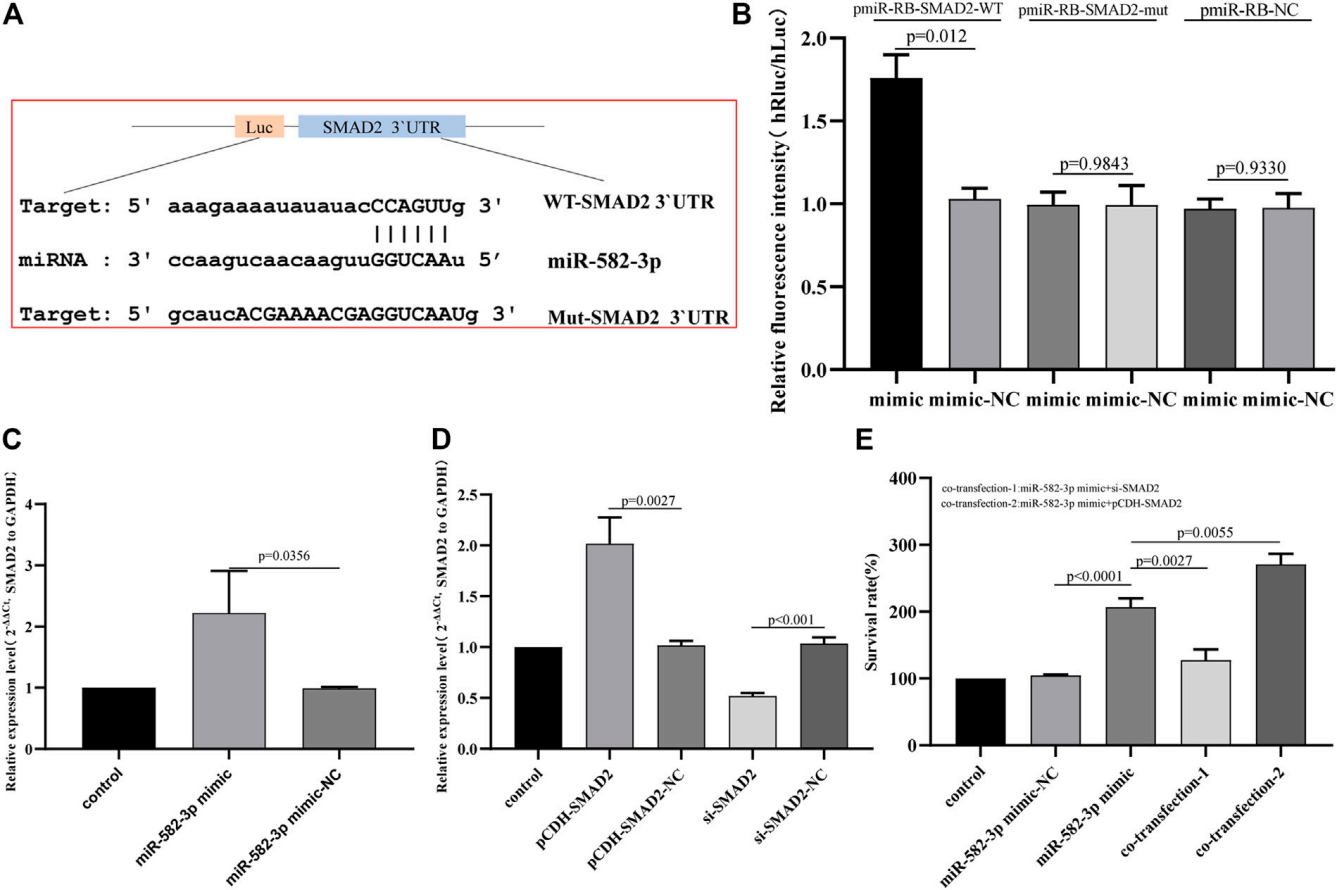
**(A)** Sequences of miR-582-3p and the SMAD2 3′UTR binding sites. **(B)** Results of the dual-luciferase assay for miR-582-3p and SMAD2. **(C)** Analysis of the effect of miR-582-3p overexpression on SMAD2 expression in HepG2 cells by qPCR. **(D)** Changes in SMAD2 expression in HepG2 cells after the addition of pCDH-SMAD2 and si-SMAD2 by qPCR. The data are shown as the mean ± standard deviation. **(E)** The effect of SMAD2 interference with miR-582-3p on the proliferation of HepG2 cells by CCK-8.

To explore the potential mechanism by which miR-582-3p regulates the behavior of HepG2 cells, we used starbase to analyze the targeting relationship between miR-582-3p and the selected hub mRNA SMAD2. Figure 7A shows the sequence of SMAD2 3′UTR binding to miR-582-3p. To confirm whether miR-582-3p directly binds SMAD2, we a performed dual-luciferase reporter gene assay and the results revealed that the fluorescence intensity of HEPG2 cells co-transfected with WT-SMAD2 3′UTR and miR-582-3p mimic was significantly higher than the control group. The fluorescence intensity of cells co-transfected with MUT-SMAD2 3′UTR and miR-582-3p mimic was similar to that of the control (Figure 7B). In addition, SMAD2 mRNA levels increased with miR-582-3p overexpression (Figure 7C). These data suggest that SMAD2 is a direct target of miR-582-3p in hepatocellular carcinoma cells.

### SMAD2 Interference Affects the Proliferation Activity of miR-582-3p on HepG2 Cells

Rescue experiments were conducted to verify whether miR-582-3p exerts biological functions by targeting SMAD2. First, the expression of SMAD2 in HepG2 cells that interfered with SMAD2 was detected by qPCR. The results revealed that pCDH-SMAD2 significantly up-regulated the expression of SMAD2, and si-SMAD2 significantly down-regulated the expression of SMAD2 (Figure 7D). The CCK-8 results showed that compared with the negative control, si-SMAD2 attenuated the effect of the miR-582-3p mimic on the proliferation activity of HepG2 cells. PCDH-SMAD2 significantly promoted the effect of the miR-582-3p mimic on HepG2 cells (Figure 7E). These results indicate that miR-582-3p regulates the proliferation of hepatocellular carcinoma cells by targeting SMAD2.

## Discussion

Abnormal expression of miRNAs, such as miR-541, miR-612, and miR-329-3p, is often detected in hepatocellular carcinoma samples and has been associated with the deterioration and pathological process of hepatocellular carcinoma (Li et al., 2020; Nagy et al.,2018; Xu et al., 2020; Liu et al., 2020; Xin et al., 2020). These results suggest that miRNAs play an important role in hepatocellular carcinoma.

In this study, 26 miRNAs were found to be abnormally expressed in hepatocellular carcinoma by miRNA sequencing, including seven up-regulated and 19 down-regulated miRNAs. These abnormally expressed miRNAs may provide valuable resources for cancer research. Kaplan-Meier curve and multivariate Cox regression analyses revealed that miR-139-5p and miR-582-3p were significantly related to the prognosis of hepatocellular carcinoma patients. The expression patterns of the 2 miRNAs in clinical tissues were completely consistent with the miRNA sequencing results. Previous studies have shown that miR-139-5p and miR-582-3p are abnormally expressed in many cancers and perform a variety of biological functions. For example, Du et al. found that KRAS mutation in response to miR-139-5p can inhibit the progression of colorectal cancer and be inhibited by Wnt signaling (Du et al.,2020). Liang et al. found that miR-139-5p inhibits the proliferation of gastric cancer cells through targeted regulation of nuclear Pre-mRNA Domain Containing 1B (Liang et al.,2020). Huang et al. found that miR-582-3p inhibited prostate cancer metastasis to bone by inhibiting TGF-β signaling (Huang et al.,2019). MiR-582-3p inhibits ovarian cancer survival and migration by targeting AKT/MTOR signaling via lncRNA TUG1 (Dai et al.,2021). In addition, Microrna-582-3p can also affect blood disorders. Li found that microrNA-582-3p negatively regulates cell proliferation and cell cycle progression in acute myeloid leukemia Targeting cyclin B2 (Li H. et al., 2019). In hepatocellular carcinoma, miR-139-5p can regulate the proliferation, migration and invasion of hepatocellular carcinoma cells by targeting different targets such as SLITRK4 (Wu et al.,2020), CCT5 (Xu et al.,2021) and ARF6 (Wu et al.,2021). However, there have been relatively few studies on hepatocellular carcinoma mediated by miR-582-3p. It has been reported that miR-582-3p can affect the progression of hepatocellular carcinoma cells by targeting circRNA HIPK3 and circRNA PTPRA, but the regulation of targeted mRNA has not been reported Therefore, miR-582-3p was selected for further study in this project.

Subsequently, we analyzed the correlation between the expression of miR-582-3p and the clinicopathological parameters of hepatocellular carcinoma patients, and found that the expression of miR-582-3p was positively correlated with TNM stage and tumor grade. High expression of miR-582-3p can promote the progression of hepatocellular carcinoma patients. Rescue experiments further confirmed that the high expression of miR-582-3p can promote the proliferation, migration and invasion of hepatocellular carcinoma cells, leading to the deterioration of hepatocellular carcinoma. These data indicate that miR-582-3p is not only an independent prognostic factor for the prognosis of hepatocellular carcinoma patients, but also a very important cancer-promoting factor in the progression of hepatocellular carcinoma. In addition, combining miRNA expression level and prognostic analysis results, we found an interesting miRNA marker: miR-378i. MiR-378i expression was down-regulated in hepatocellular carcinoma, and patients with high expression of miR-378i have a poor prognosis. This may be because the expression pattern of miR-378i itself in hepatocellular carcinoma is not correlated with survival data, only the low expression of miR-378i is correlated with tumorigenesis, while the high expression of miR-378i is correlated with tumor progression. Similar studies have been reported in the past, such as bidirectional regulation of LINC01235 in breast cancer, and different expression patterns of DAPLE gene in different stages of colon cancer (Li et al., 2021; Aznar et al.,2015). It may also be related to the immune escape mechanism of hepatocellular carcinoma cells. However, all these needs further research.

MiRNA is a noncoding RNA that generally regulates gene expression by combining with its target mRNA to mediate tumor growth, angiogenesis and metastasis (Ali Syeda et al.,2020; Saliminejad et al.,2019). In this study, we predicted the targeted mRNAs of miR-582-3p through miRwalk and starbase, and then the key pairs miR-582-3p/SMAD2 were screened by WGCNA, qPCR and Pearson correlation analysis. qPCR and dual luciferase experiments confirmed that miR-582-3p directly targets SMAD2. Rescue-of-function experiments confirmed that interference with the expression of SMAD2 can regulate the effect of miR-582-3p on the proliferation activity of HepG2 cells. At the same time, interference with SMAD2 also affected the expression of miR-582-3p in HepG2 cells. These dates indicate that miR-582-3p can regulate the proliferation of HepG2 and other biological processes by targeting SMAD2.

SMAD family member 2 (SMAD2), a member of SMAD, is an intracellular signal transduction and transcription regulator downstream of the TGF-β signaling pathway that is activated by TGF-β and the activin receptors TβRI and ActRIIB through carboxyl terminal phosphorylation (Zhou et al.,2020; Kamato and Little, 2020). According to reports, SMAD2 has a unique role in cell biology and is involved in tissue differentiation, development, inflammation, and tumorigenesis (Tang et al.,2018). The abnormal expression of SMAD2 can directly inhibit the activation state of the TGF-β signaling pathway, thereby freeing tumor cells from growth inhibition (Hu et al., 2018). At present, studies have confirmed that SMAD2 is the target gene of some specific miRNAs in other tumors. For example, Li et al. pointed out that in non-small-cell lung cancer, miR-433 can inhibit tumor progression by targeting Smad2 (Li J. et al.,2019). Zhou et al. found that miR-200b-3p can target SMAD2 to promote the progression of melanoma (Zhou et al.,2020). Hu et al. found that miR-484 inhibits the proliferation and epithelial-mesenchymal transition of cervical cancer cells by targeting ZEB1 and SMAD2 (Hu et al.,2021).

In this study, we used miRNA sequencing and an integrated bioinformatics approach to explore hub miRNA-mRNA interactions that are highly associated with hepatocellular carcinoma development. Our data indicated that miR-582-3p is closely related to the overall survival rate of hepatocellular carcinoma patients and directly combines with SMAD2 to regulate its expression. In addition, the expression of miR-582-3p is positively correlated with the clinicopathological parameters of hepatocellular carcinoma patients, and its high expression promotes the proliferation, migration and invasion of hepatocellular carcinoma cells. Interfering with the expression of SMAD2 can regulate the expression of miR-582-3p and its proliferative activity on hepatocellular carcinoma cells.

## Conclusion

Based on all the analysis results, we speculate that miR-582-3p may regulate hepatocarcinogenesis by targeting SMAD2. However, this inference needs to be further confirmed by experimental data before these results can be applied to explore clinical biomarkers or therapeutic targets.

## Data Availability

The datasets presented in this study can be found in online repositories. The names of the repository/repositories and accession number(s) can be found in the article/Supplementary Material.
