# Supplementary figures and images for: Use of miRNA Sequencing to Reveal Hub miRNAs and the Effect of miR-582-3p/SMAD2 in the Progression of Hepatocellular Carcinoma
Source: Front Genet. 2022 Mar 21;13:819553. doi: 10.3389/fgene.2022.819553 (PMC8977860; doi:10.3389/fgene.2022.819553)

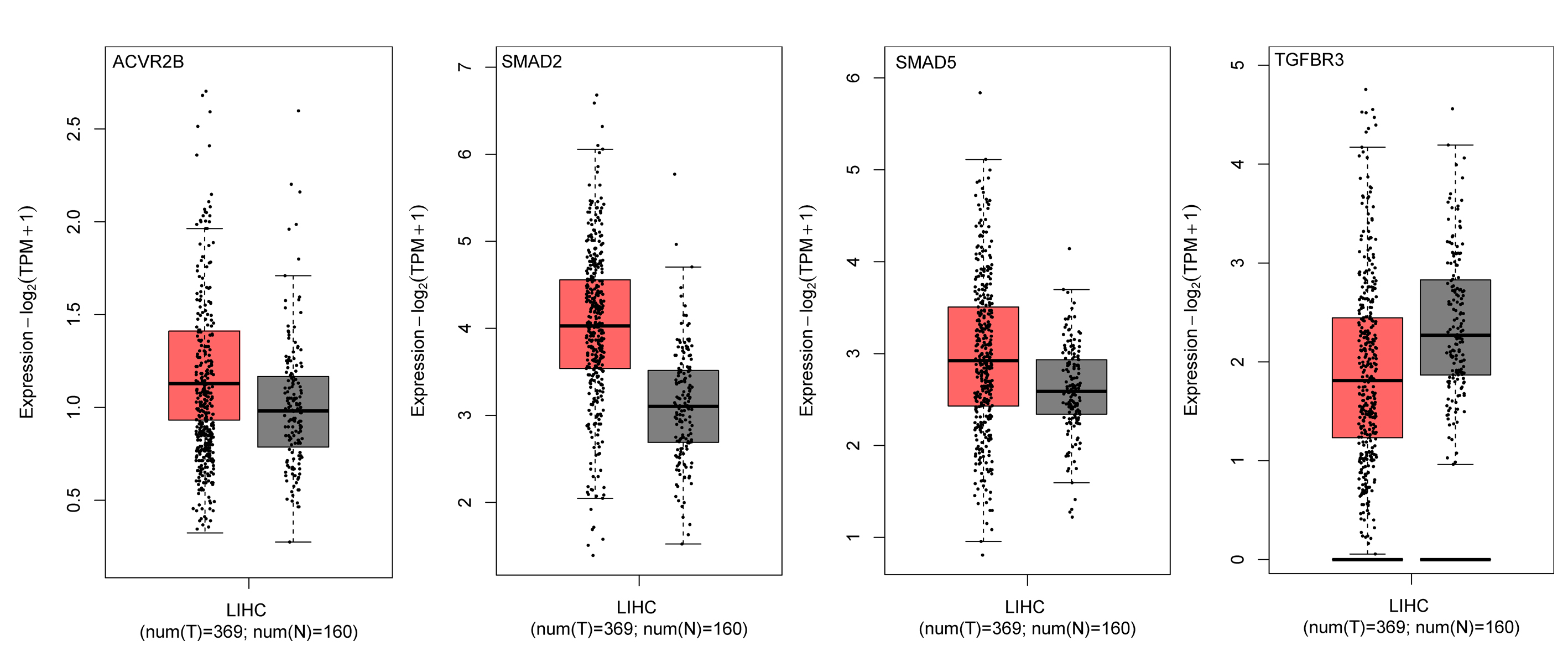

Supplement: Supplementary file 1 [file Image1.JPEG]
