# Supplementary material for: Use of miRNA Sequencing to Reveal Hub miRNAs and the Effect of miR-582-3p/SMAD2 in the Progression of Hepatocellular Carcinoma
Source: Front Genet. 2022 Mar 21;13:819553. doi: 10.3389/fgene.2022.819553 (PMC8977860; doi:10.3389/fgene.2022.819553)
Supplement: Supplementary file 2 [file DataSheet1.zip › supplemental material/supplemental material 2.docx]

Supplemental material 2. The sequences of primers.

| **Gene symbol** | **Primer** | **Sequences (5’-3’)** |
| --- | --- | --- |
| ACVR2B | Forward primer | TCAACTGCTACGATAGGCAG |
|  | Reverse primer | AGAAGTTGCCTTCACAGCA |
| SMAD2 | Forward primer | TTACCATACCAAGCACTTGC |
|  | Reverse primer | AAAGGCCTGTTGTATCCCA |
| SMAD5 | Forward primer | TGGGATTACAGGACTTGACC |
|  | Reverse primer | CATTTGACACAAATCTTCGGAG |
| TGFBR3 | Forward primer | CTGCAGGAGGTTTGTTCAG |
|  | Reverse primer | ATTTAATGAGCTTGCGTCACC |
| GAPDH | Forward primer | CCTCCTGTTCGACAGTCAG |
|  | Reverse primer | CCCATACGACTGCAAAGAC |
